# Elevated thyroid hormones caused by high concentrate diets participate in hepatic metabolic disorders in dairy cows

**DOI:** 10.5713/ab.21.0397

**Published:** 2022-01-05

**Authors:** Qu Chen, Chen Wu, Zhihao Yao, Liuping Cai, Yingdong Ni, Shengyong Mao

**Affiliations:** 1 Key Laboratory of Animal Physiology and Biochemistry, Nanjing Agricultural University, Nanjing 210095, China; 2 Laboratory of Gastrointestinal Microbiology, Jiangsu Key Laboratory of Gastrointestinal Nutrition and Animal Health, College of Animal Science and Technology, Nanjing Agricultural University, Nanjing 210095, China

**Keywords:** Glucose Metabolism, High Concentrate Diets, Lactating Dairy Cows, Lipid Metabolism, Thyroid Hormones

## Abstract

**Objective:**

High concentrate diets are widely used to satisfy high-yielding dairy cows; however, long-term feeding of high concentrate diets can cause subacute ruminal acidosis (SARA). The endocrine disturbance is one of the important reasons for metabolic disorders caused by SARA. However, there is no current report about thyroid hormones involved in liver metabolic disorders induced by a high concentrate diet.

**Methods:**

In this study, 12 mid-lactating dairy cows were randomly assigned to HC (high concentrate) group (60% concentrate of dry matter, n = 6) and LC (low concentrate) group (40% concentrate of dry matter, n = 6). All cows were slaughtered on the 21st day, and the samples of blood and liver were collected to analyze the blood biochemistry, histological changes, thyroid hormones, and the expression of genes and proteins.

**Results:**

Compared with LC group, HC group showed decreased serum triglyceride, free fatty acid, total cholesterol, low-density lipoprotein cholesterol, increased hepatic glycogen, and glucose. For glucose metabolism, the gene and protein expression of glucose-6-phosphatase and phosphoenolpyruvate carboxykinase 1 in the liver were significantly up-regulated in HC group. For lipid metabolism, the expression of sterol regulatory element-binding protein 1, long-chain acyl-CoA synthetase 1, and fatty acid synthase in the liver was decreased in HC group, whereas carnitine palmitoyltransferase 1α and peroxisome proliferator activated receptor α were increased. Serum triiodothyronine, thyroxin, free triiodothyronine (FT3), and hepatic FT3 increased in HC group, accompanied by increased expression of thyroid hormone receptor (THR) in the liver.

**Conclusion:**

Taken together, thyroid hormones may increase hepatic gluconeogenesis, β-oxidation and reduce fatty acid synthesis through the THR pathway to participate in the metabolic disorders caused by a high concentrate diet.

## INTRODUCTION

To satisfy energy requirements for maintaining a high milk yield, excessive-high concentrate diets are used to feed dairy cows [1]. However, over-consumption of high concentrate diets can induce subacute ruminal acidosis (SARA), which leads to a series of metabolic diseases, including loss of appetite, diarrhea, laminitis, and inflammation, eventually threatening animal health and reducing production performance [2–4].

The liver as a metabolic organ is important for animal health and production performance. Hepatic gluconeogenesis is responsible for maintaining an adequate glucose supply to the mammary glands [5]. Glucose-6-phosphatase (G6PC) and phosphoenolpyruvate carboxykinase 1 (PCK1) is the rate-limiting enzymes involved in hepatic gluconeogenesis [6]. Hepatic lipid metabolism is important for milk quality [7]. Sterol regulatory element-protein 1 (*SREBP1*), fatty acid synthase (*FAS*), and long-chain acyl CoA synthetase 1 (*ACSL1*) are important genes for *de novo* fatty acid synthesis [8,9]. Carnitine palmitoyltransferase 1A (CPT1α) and peroxisome proliferator activated receptor alpha (PPARα) are important for dairy cows to regulate the entry of fatty acids into the mitochondria for β-oxidation [10]. Abnormal glucose and lipid metabolisms in the liver can cause diseases and decrease milk yield and quality in ruminants [9,11].

Hormones play important roles in glucose and lipid metabolisms during the lactation period [12]. Studies found that high concentrate diets can result in the abnormal secretion of some hormones [7,13]. A recent study reported that blood thyroid hormones can be affected by the diet in dairy cows [14]. Thyroid hormones are widely involved in metabolism, including glycometabolism and lipid metabolism [15, 16]. Thyroxin (T4) and triiodothyronine (T3) are two major types of thyroid hormones, while T3 is the active form of thyroid hormone with a high affinity to thyroid hormone receptors (THR). The circulated thyroid hormones are majorly combined with proteins, however, only free triiodothyronine (FT3) can enter cells by combining with their receptors [17]. The THR acts as a ligand-dependent transcription factor to regulate genes involved in glucose and lipid metabolisms [18]. This study was undertaken to evaluate whether the thyroid hormones and THR participate in hepatic metabolic disorders in dairy cows fed a high concentrate diet.

## MATERIALS AND METHODS

### Ethics statement

Animal procedures were permitted by the Institutional Animal Care and Use Committee (IACUC) of Nanjing Agricultural University according to the Guidelines for Experimental Animals of the Ministry of Science and Technology (2006, Beijing, China).

### Experimental animals

Twelve healthy Chinese Holstein cows (mean body weight, 651±54 kg) during mid-lactation (mean milk yield, 17.43± 4.04 kg/d) were raised in a dairy farm (Jiangsu Province, China) for the study. All the cows were in their second or third parity when the liver tissues were collected. These cows were kept in free-stall housing in the same cubicle partition of the barn during the experimental period. After a week of prefeeding, the cows were randomly assigned to LC and HC groups. LC group received low concentrate diet (40% of dry matter) (n = 6), and HC group received high concentrate diet (60% of dry matter) (n = 6) for 3 weeks. The ingredients in the diets and the nutritional composition are shown in Table 1. Cows were free to access freshwater throughout the experimental time.

### Samples collection

All cows were fasted for 12 h, with free access to water. Then all animals were slaughtered with the application of electrical stunning. The jugular vein was cut, and blood samples were collected using 5 mL tubes without anticoagulants. The liver tissues at the same site were collected and soaked in 4% paraformaldehyde for hematoxylin and eosin (HE) and periodic acid-Schiff (PAS) staining. The remaining liver tissues were frozen immediately in liquid nitrogen and stored at −80°C for subsequent extraction of RNA and proteins.

### Staining for periodic acid-Schiff

After being fixed with a 4% paraformaldehyde for 48 h at 4°C, dehydrated with ethanol the slides were embedded with paraffin. The paraffin-embedded slides were deparaffinized, hydrated with distilled water, and oxidized for 10 min at 40°C in a 0.5% periodic acid solution. The slides were washed with distilled water, immersed in Schiff reagent, washed in distilled water, counterstained with Mayer’s hematoxylin, washed in distilled water, dehydrated in a gradient of ethanol, and ultimately sealed using a synthetic mounting medium.

### Measurement of biochemical parameters in serum and liver

All serum biochemical parameters, including glucose, triglyceride (TG), free fatty acid (FFA), total cholesterol (TC), low-density lipoprotein cholesterol (LDL-C), and high-density lipoprotein cholesterol (HDL-C) were measured using an automatic biochemical analyzer (7020; HITACHI, Tokyo, Japan) with the respective assay kits following the manufacturer’s instructions (ShinoTest Corp., Tokyo, Japan). Hepatic glycogen and FFA were determined using commercial kits, provided by Jiancheng Co. Ltd (Nanjing, China). Hepatic TG was determined using a commercial kit, provided by Applygen Co. Ltd (Beijing, China). Hepatic glucose was determined using a commercial kit, provided by Comin Co. Ltd (Suzhou, China). All kits were strictly following the manufacturer’s instructions.

### Determinations of hormones in serum and liver

The method for extracting hepatic FT3 was described by Beckett et al [19]. The levels of T3, T4, and FT3 were determined using commercial radioimmunoassay kits, provided by North institute of Biotechnology Co. Ltd (Beijing, China). All experiments were conducted following the manufacturer’s instructions strictly.

### RNA isolation, cDNA synthesis, and real-time polymerase chain reaction

Total RNA was extracted from 40 mg of liver tissue using the TRIzol reagent (Sangon, Shanghai, China). The concentration and quality of the RNA were measured with a NanoDropND-1000 Spectrophotometer (Thermo Fisher Scientific, Madison, WI, USA). The first-strand cDNA was synthesized using 200 ng of the total RNA template using the All-in-One First-Strand cDNA Synthesis SuperMix for qPCR kit (Transgene Co., Beijing, China). Two microliters of diluted cDNA (1:40, v/v) were used for real-time polymerase chain reaction (PCR), which was performed in an Mx3000P (Stratagene, Palo Alto, CA, USA). The quantitative PCR (qPCR) was performed using the SYBR Green qPCR SuperMix (Transgene Co., China) on an ABI 7300 Real-Time PCR System (Applied Biosystems, Foster City, CA, USA). The PCR protocol was described in the manual: denaturing at 95°C for 30 s, then 40 cycles at 95°C for 5 s, and 60°C for 30 s. Glyceraldehyde phosphate dehydrogenase, which is not affected by the experimental factors, was chosen as the reference gene. The primers were designed using Premier 6.0 (Premier Biosoft International, San Francisco, CA, USA) (Table 2), and synthesized by Tsingke Company (Nanjing, China). The method of 2−Ct was used for the relative quantification.

### Western blotting analysis

One hundred milligrams of the frozen liver were minced and homogenized in 1 mL of ice-cold radioimmunoprecipitation assay lysis buffer containing the protease inhibitor cocktail complete ethylenediaminetetraacetic acid-free (Roche, Penzberg, Germany). The homogenates were centrifuged at 12,000 g for 20 min at 4°C and then the supernatant fraction was collected. The protein concentration was determined using the bicinchoninic acid protein assay kit (Thermo, USA). Forty micrograms of protein extract from each sample were then loaded into 10% sodium dodecyl sulfate-polyacrylamide gel electrophoresis gels, and the separated proteins were transferred onto nitrocellulose membranes (BioTrace; Pall Corp., New York, NY, USA). Membranes were blocked for 2 h at room temperature in blocking buffer and then incubated with the following primary antibodies: rabbit-anti-PCK1 (1:1,000; 12940S; CST, Boston, MA, USA), rabbit-anti-SREBP1 (1:1,000; 14088-1-AP; Proteintech, Wuhan, Hubei, China), rabbit-anti-FAS (1:1,000; BS6050; Bioworld, Nanjing, Jiangsu, China), rabbit-anti-G6PC (1:1,000; ab83690; Abcam, Cambridge, UK), rabbit-anti-CPT1α (1:1,000; 15184-1-AP; Proteintech, China), rabbit-anti-PPARα (1:1,000; 15540-1-AP; Proteintech, China), rabbit-anti-THRB (1:1,000; AF8157; Beyotime, Shanghai, China), rabbit-anti-iodothyronine deiodinase 2 (DIO2) (1:1,000; bs-3673r; Bioss, Beijing, China), rabbit-anti-actin beta (1:10,000; KC-5AO8; Kangchen bio-tech, Shanghai, China) in dilution buffer overnight at 4°C. After several washes in Tris-buffered-saline with Tween, membranes were incubated with goat anti-rabbit HRP-conjugated secondary antibodies (1:10,000; BS13278; Bioworld, China) in dilution buffer for 2 hours at room temperature. Finally, the blot was washed and detected by enhanced chemiluminescence using the LumiGlo substrate (Super Signal West Pico Trial Kit; Pierce, Thermo Fisher Scientific, USA) and the signals were recorded by an imaging system (Bio-Rad, Hercules, CA, USA) and analyzed by Image J software.

## RESULTS

### Effect of high concentrate diets on ruminal pH value and serum biochemistry

**Figure 1:**
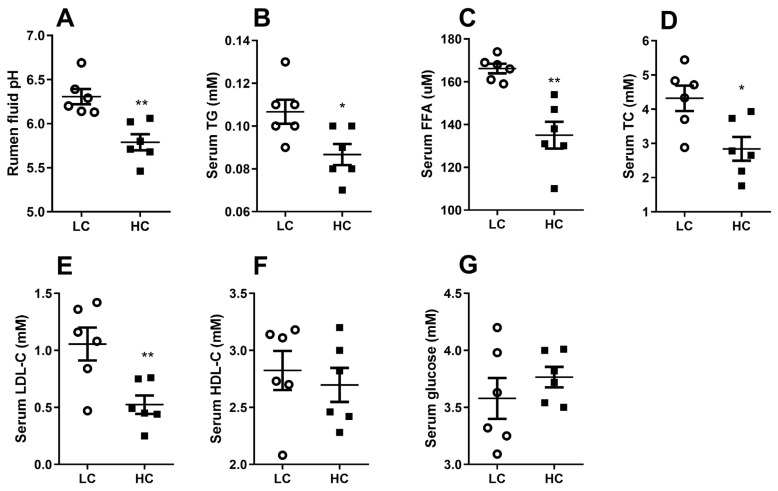
Effects of low or high concentrate (LC or HC) diets on ruminal pH and serum biochemical parameters at the 21st day. (A) Ruminal fluid pH. (B) The levels of serum triglyceride (TG). (C) The levels of serum free fatty acid (FFA). (D) The levels of serum total cholesterol (TC). (E) The levels of serum low-density lipoprotein cholesterol (LDL-C). (F) The levels of serum high-density lipoprotein cholesterol (HDL-C). (G) The levels of serum glucose. Values are mean±standard error of the mean. The data were analyzed by independent-samples T test using the Compare Means of SPASS 19.0 for Windows (StaSoft Inc, Tulsa, OK, USA). * p<0.05, ** p<0.01 (LC vs HC).

As is shown in Figure 1A, the pH of ruminal fluid remarkably decreased in HC group on the 21st day (p<0.01). The levels of serum TG, FFA, TC, and LDL-C were significantly decreased in HC group (Figure 1B-E) (p<0.05), whereas there was no significant difference in serum glucose and HDL-C concentrations (Figure 1F, 1G) (p>0.05).

### Effect of high concentrate diets on liver histology and biochemistry

**Figure 2:**
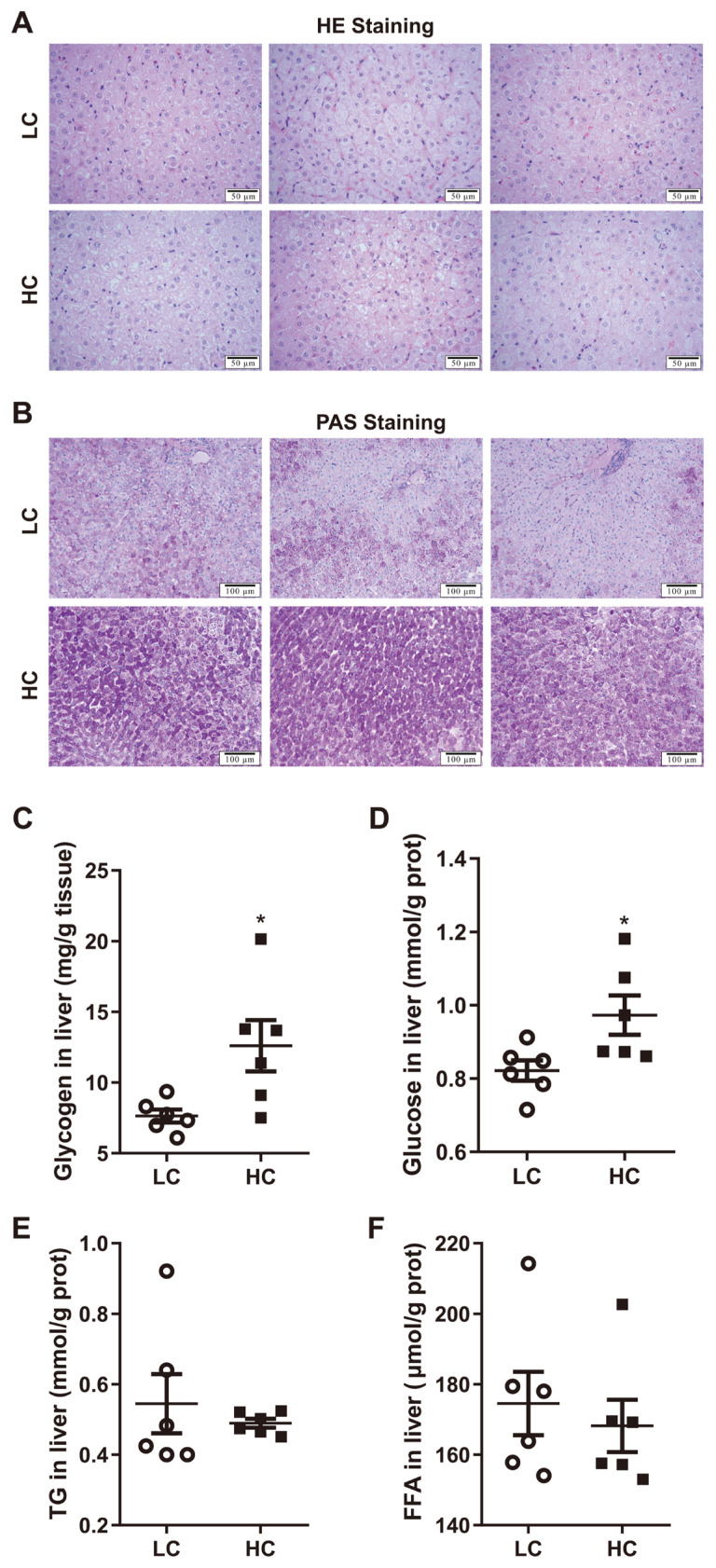
Effects of low or high concentrate (LC or HC) diets on histological changes and biochemical parameters in the liver. Photomicrograph of hematoxylin and eosin (HE) (A) and periodic acid-Schiff (PAS) (B) staining in cow liver, these pictures are from different cows. (C) The levels of hepatic glycogen. (D) The levels of hepatic glucose. (E) The levels of hepatic triglyceride (TG). (F) The levels of hepatic free fatty acid (FFA). Values are mean±standard error of the mean. The data were analyzed by independent-samples T test using the compare means of SPASS 19.0 for Windows (StaSoft Inc, Tulsa, OK, USA). * p<0.05 (LC vs HC).

There was no obvious lipid deposition, inflammation or damages in the liver between LC and HC groups demonstrated by HE staining sections (Figure 2A). The levels of hepatic TG and FFA were not significantly changed by high concentrate diets (Figure 2E, 2F) (p>0.05). However, PAS staining showed that high concentrate diets significantly promoted hepatic glycogen accumulation (Figure 2B). Biochemical detection also confirmed an increase in the levels of hepatic glycogen and glucose in dairy cows fed a high concentrate diet (Figure 2C, 2D) (p<0.05).

### Expression of genes and proteins related to glucose metabolism in the liver

**Figure 3:**
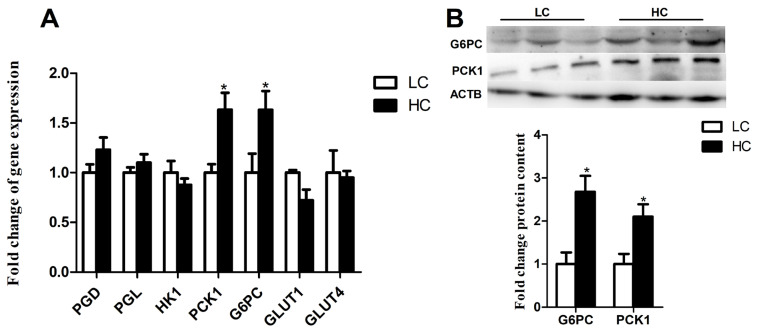
Expression of genes and proteins related to glucose metabolism in the liver. (A) Expression of genes related to glucose metabolism in the liver. (B) Expression of proteins related to gluconeogenesis in the liver. The data were analyzed by independent-samples T test using the compare means of SPASS 11.0 for Windows (StaSoft Inc, Tulsa, OK, USA). Values are mean±standard error of the mean. * p<0.05 (LC vs HC).

Real-time PCR showed that the expression of PCK1 and G6PC in the liver were significantly increased in HC group (Figure 3A) (p<0.05). Other genes including phosphogluconate dehydrogenase (*PGD*), succinate dehydrogenase complex subunit D (*PGL*), hexokinase 1 (*HK1*), solute carrier family 2 member 1 (*GLUT1*), and solute carrier family 2 member 4 (*GLUT4*) did not show a significant difference (Figure 3A) (p>0.05). Following the genes expression, the expression of *G6PC* and *PCK1* were significantly increased in HC group at the protein level (Figure 3B) (p<0.05).

### Expression of genes and proteins related to lipid metabolism in the liver

**Figure 4:**
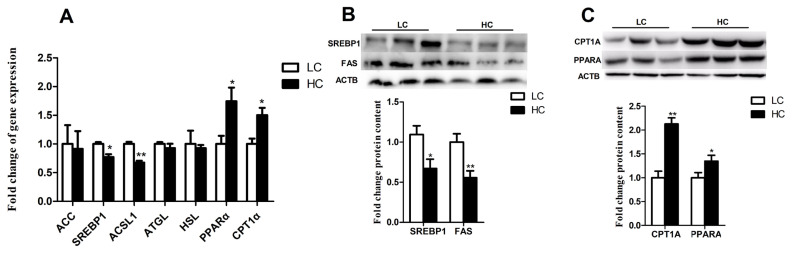
Expression of genes and proteins related to lipid metabolism in the liver. (A) Expression of genes related to lipid metabolism in the liver. (B) Expression of proteins related to fatty acid synthesis in the liver. (C) Expression of proteins related to fatty acid β-oxidation in the liver. The data were analyzed by independent-samples T test using the compare means of SPASS 11.0 for Windows (StaSoft Inc, Tulsa, OK, USA). Values are mean±standard error of the mean. * p<0.05, ** p<0.01 (LC vs HC).

The mRNA expression of SREBP1 and ACSL1 were significantly decreased in cows fed a high concentrate diet (Figure 4A) (p<0.05), whereas PPARα and CPT1α were significantly increased (Figure 4A) (p<0.05). However, other genes including acetyl-CoA carboxylase alpha (*ACC*), hormone-sensitive lipase (*HSL*), and adipose triglyceride lipase (*ATGL*) did not alter between LC and HC groups (Figure 4A) (p>0.05). Consistent with the mRNA expression, the protein expression of SREBP1 and FAS were significantly decreased (Figure 4B) (p<0.05), CPT1α and PPARα were significantly increased in HC group (Figure 4C) (p<0.05).

### Effect of high concentrate diets on thyroid hormones levels in serum and liver

**Figure 5:**
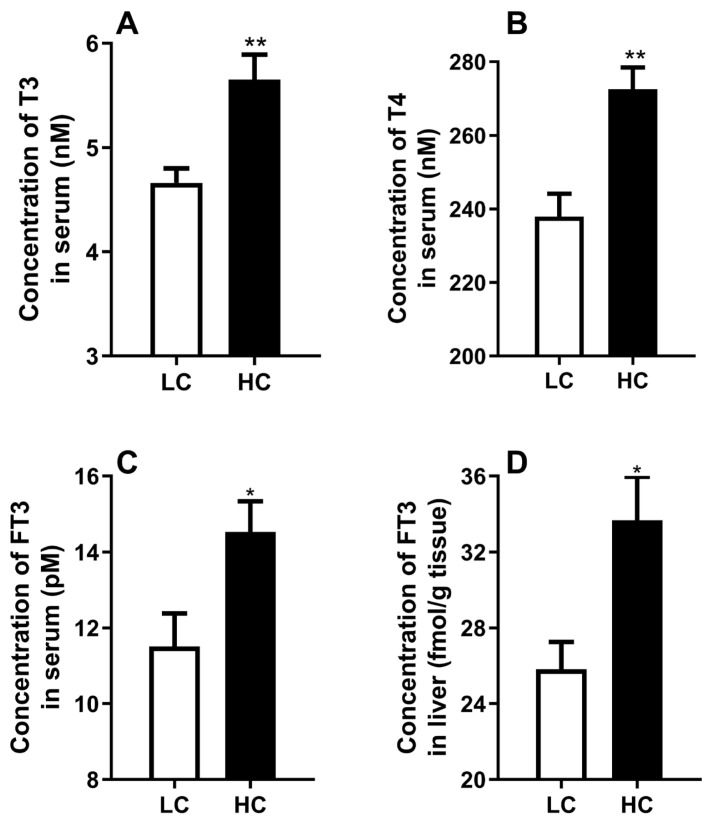
Effects of low or high concentrate diet on hormones. (A) The levels of serum cortisol. (B) The levels of serum insulin. (C) The levels of serum T4. (D) The levels of serum T3. (E) The levels of serum free triiodothyronine (FT3). (F) The levels of hepatic FT3. The data were analyzed by independent-samples T test using the compare means of SPASS 11.0 for Windows (StaSoft Inc, Tulsa, OK, USA). Values are mean±standard error of the mean. * p<0.05, ** p<0.01 (LC vs HC).

Compared to LC group, the cows in HC group showed a significant increase in serum T3, T4, FT3, and hepatic FT3 (Figure 5) (p<0.05).

### Effect of high concentrate diets on the expression of THR in the liver

**Figure 6:**
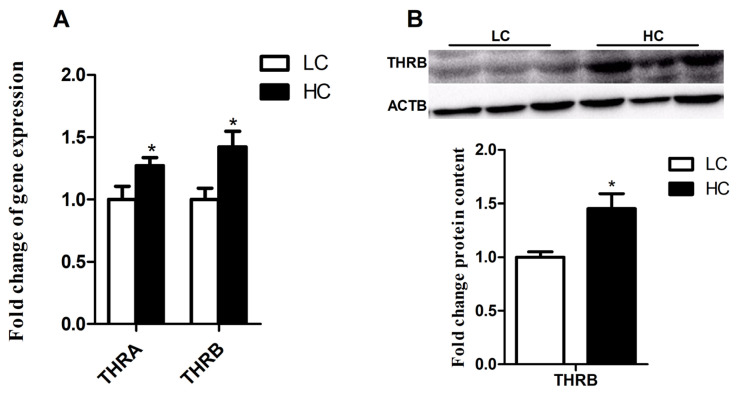
The expression of genes and protein related to thyroid hormone receptor (THR) in the liver. (A) Gene expression of *THRA* and *THRB* in the liver. (B) Protein expression of THRB in the liver. The data were analyzed by independent-samples T test using the compare means of SPASS 11.0 for Windows (StaSoft Inc, Tulsa, OK, USA). Values are mean±standard error of the mean. * p<0.05 (LC vs HC).

The result of qPCR showed that the expression of THRA and THRB was significantly up-regulated by high concentrate diets (Figure 6A) (p<0.05). Similarly, the protein expression of THRB also increased in HC group (Figure 6B) (p<0.05).

### Effect of high concentrate diets on iodothyronine deiodinases in the liver

**Figure 7:**
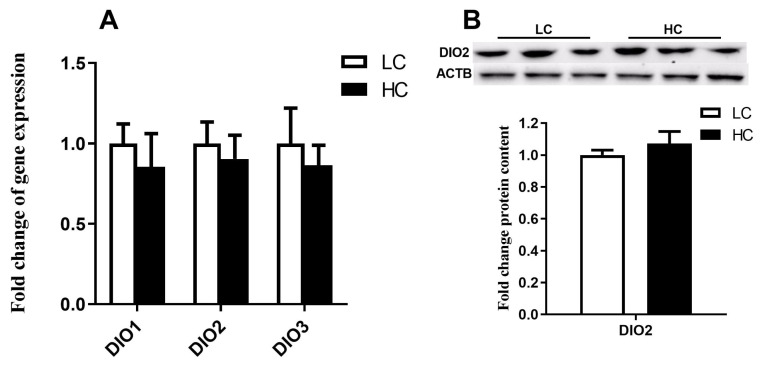
The expression of genes and protein related to iodothyronine deiodinases (*DIO*) in the liver. (A) Gene expression of *DIO1*, *DIO2*, and *DIO3* in the liver. (B) Protein expression of DIO2 in the liver. The data were analyzed by independent-samples T test using the compare means of SPASS 11.0 for Windows (StaSoft Inc, Tulsa, OK, USA). Values are mean±standard error of the mean. * p<0.05 (LC vs HC).

Three isoforms of the iodothyronine deiodinases, including DIO1, DIO2, and DIO3 were detected by qPCR, however, these genes did not show an obvious difference between LC and HC groups (Figure 7A) (p>0.05). Western blot analysis also confirmed that DIO2 was unaltered by high concentrate diets at the protein level (Figure 7B) (p>0.05).

## DISCUSSION

Liver is an important organ in regulating glycometabolism and lipid metabolism [11,20]. Previous studies reported that high concentrate diets can induce TG accumulation, inflammation, and tissue damages in ruminants [9,21]. Although these changes were not shown in HE staining sections, we observed remarkable glycogen deposition in HC group via PAS staining. Biochemical analysis also confirmed that hepatic glucose and glycogen concentrations significantly increased in HC group. Unlike non-ruminant mammals, ruminants primarily depend on hepatic gluconeogenesis as their source of glucose to meet their energy demand [22]. Dong et al [9] found that high concentrate diets significantly increased the enzyme activities involved in gluconeogenesis, including G6PC and PCK1, whereas there were no obvious changes in the enzyme activities involved in glycolysis. It is highly consistent with our data, high concentrate diets significantly up-regulated gluconeogenic genes and proteins expression in HC group. Hepatic gluconeogenesis is essential to meet glucose requirements for milk production by mobilization of endogenous glucogenic substrates [5,23]. Hence, feeding high concentrate diets can promote milk yield in a certain period via enhanced hepatic gluconeogenesis [9]. However, several studies have found that long-term feeding of high concentrate diets can induce metabolic disorders which are related to abnormal hormones levels, including cortisol, leptin, and insulin [7,24]. Because these altered hormones could not fully explain the complex metabolic disorders caused by high concentrate diets, we hypothesized that some hormones involved in metabolic disorders were ignored. Graugnard et al [25] reported that high-starch diets induced a marked increase in the gene expression of thyroid hormone-responsive (SPOT14 homologue, rat) (THRSP) in longissimus lumborum of cattle, suggesting thyroid hormones may be involved in [25]. It has been known that thyroid hormones play important roles in glycometabolism and lipid metabolism. However, fewer studies were conducted to examine the roles of thyroid hormones in metabolic abnormalities caused by high concentrate diets. Most studies considered that thyroid hormones can promote gluconeogenesis. Researchers found that thyroid hormones can directly elevate the expression of gluconeogenesis rate-limiting enzymes PCK1 and G6PC [26,27]. THR can enhance hepatic gluconeogenesis not only by directly promoting PCK1 and G6PC expression but also by deacetylation of the master gluconeogenic transcription factor forkhead box O1 (FoxO1) [28]. The animal experiment also showed that injecting T4 significantly increased milk yield in dairy cows, accompanied by enhanced hepatic gluconeogenesis [29]. Therefore, in our study, elevated thyroid hormones levels and hepatic THR expression can explain the increase of glucose and glycogen in the liver by enhancing hepatic gluconeogenesis.

In recent years, the decrease in milk fat rate caused by a high concentrate diet has attracted the attention of many researchers [30–32]. TG and FFA, the main precursors of milk fat, have a close relationship to milk fat content [33]. Our data showed a significant decrease in serum FFA and TG in dairy cows fed a high concentrate diet. As one of the most important organs involved in lipid metabolism, the liver plays an important role in regulating blood TG and FFA. Jiang et al [34] employed a comparative proteomic approach to investigate the effect of high concentrate diets on the hepatic metabolism in lactating dairy goats. They found high concentrate diets enhanced hepatic TG synthesis and accumulation, and reduced TG output, eventually reducing milk fat rate. However, Xu et al [11] found that a high concentrate diet decreased TG synthesis and increased fatty acids decomposition, accompanied by reduced plasma TG and non-esterified fatty acid, eventually reducing milk fat rate. In our data, the genes or proteins related to fatty acids synthesis, including SREBP1, ACSL1, and FAS, significantly reduced in HC group; whereas the expression of genes and proteins related to β-oxidation, including CPT1α and PPARα, significantly increased. These different findings can be attributed to the changes in thyroid hormones. T3 can not only increase hepatic lipid deposition by upregulating lipogenesis, but also promote hepatic lipolysis by enhancing β-oxidation [28]. Therefore, the regulatory effect of thyroid hormones on hepatic lipid metabolism is complex, it may be related to the dose and time of thyroid hormones treatment. In conclusion, whether hepatic lipid deposition or fatty acids oxidation induced by thyroid hormones can reduce blood TG and FFA concentrations, the result is a depression in milk fat.

Tissue sensitivity to thyroid hormones can be altered by iodothyronine deiodination and changes in the expression of nuclear receptors for thyroid hormones [35]. Our data showed that THRA and THRB were highly increased in HC group. Compared to T4, T3 has more intrinsic biological activity due to its high affinity for THR [36]. The result of increased serum T3, T4, FT3, and hepatic FT3 further confirmed that thyroid hormones may change glucose metabolism and lipid metabolism through the THR pathway. Three isoforms of iodothyronine deiodinase regulate the local and systemic activity of thyroid hormones. DIO1 and DIO2 deiodinate the outer ring of T4 to yield active hormone T3; whereas DIO3 converts T4 to the inactive reverse T3 (rT3) or converts active T3 to the inactive diiodothyronineto (T2). The expression of DIO1, DIO2, and DIO3 in the liver did not show a significant difference between LC and HC groups, demonstrating that high concentrate diets did not change the expression of iodothyronine deiodinase. Taken together, the changes of thyroid hormones enrich the endocrine theory of metabolic disorders caused by high concentrate diets, which helps to provide a new direction for future research.

## CONCLUSION

In this study, dairy cows fed a high concentrate diet showed decreased serum TG, FFA concentrations, and increased hepatic glycogen deposition. These changes can attribute to enhanced hepatic gluconeogenesis, β-oxidation, and suppressed fatty acids synthesis. Elevated thyroid hormones in serum and liver may change hepatic glucose and lipid metabolisms via the activation of the THR pathway. Our study reports for the first time that the changes in thyroid hormones will further enrich the mechanisms of metabolic disorders induced by high concentrate diets. Moreover, it is also of reference value for the application of thyroid hormones. Although thyroid hormones can improve energy metabolism and milk yield in dairy cows within a period, attention should be paid to the long-term application that may cause decreased milk quality and metabolic disorders.

## Figures and Tables

**Table 1:** The ingredients in the diets and the nutritional composition

Items	Low concentrate diets	High concentrate diets
Ingredient (% DM)		
Corn	19.4	24.92
Soybean meal	13.5	13.48
DDGS	3.8	5.91
Stone meal	0.8	1.48
CaHCO_3_	1.1	0.92
NaCl	0.4	0.37
Premix^1)^	1	0.92
Barley	/	12
Silage corn	12	6
Alfalfa hay	24	17
Oat hay	24	17
Nutritional composition^2)^ (% of DM)		
Crude protein	16.12	16.2
Ca	1.18	1.15
P	0.51	0.53
Neutral detergent fiber	29.92	27.75
Non-fiber carbohydrates	42.34	44.47
Ash	4.87	4.56
Ether extract	3.05	3.02
NE_L_ (Mcal/kg of DM)	1.64	1.66

DM, dry matter; DDGS, distillers dried grains with solubles; NE_L_, net energy for lactation.

1) The premix contained Vit A, 1,900 ku/kg; Vit D, 250 ku/kg; Vit E, 3,000 mg/kg; niacin, 4,000 mg/kg; Cu, 1,200 mg/kg; Fe, 525 mg/kg; Zn, 13,000 mg/kg; Mn, 5,500 mg/kg; I, 170 mg/kg; Co, 50 mg/kg; Se, 27 mg/kg.

2) Calculated values.

**Table 2:** Primer sequences of the target genes

Gene	Sequence number	Primer sequences (5′-3′)	Length (bp)
*SREBP1*	NM_001113302.1	F: CATCAGCTCCAGCATGGCT R: TGGGTAGGGGTTTCTCGGA	214
*G6PC*	NM_001076124.2	F: ATGTTGTGGTTGGGATTCTGG R: CACCTTCGCTTGGCTTTCTC	151
*PCK1*	NM_174737.2	F: GCCGTGAGGAGTTTCGTG R: TGATGATGACCGTCTTGCT	120
*ACC*	NM_174224	F: AGCTGAATTTTCGCAGCAAT R: GGTTTTCTCCCCAGGAAAAG	117
*ACSL1*	NM_001076085.1	F: TCGGAACTGAAGCCATCACC R: GCCTCGTTCCAGCAGATCAC	144
*ATGL*	NM_001046005.2	F: TCTGCCTGCTGATTGCTATG R: GGCCTGGATAAGCTCCTCTT	98
*HSL*	NM_001080220.1	F: ATTGCCGACTTCCTACGAGA R: AGTCCGATGGAGATGGTCTG	113
*GLUT1*	NM_174602.2	F: GGGCATGTGCTTCCAGTATGTG R: TGTCTCGGGAACTTTGAAGTAGGTG	106
*GLUT4*	NM_174604.1	F: TGCGTCTCCAGTTCCTAAGACAAG R: AAGGACCAAGGTCCCAGTGA	153
*HK1*	NM_001012668.2	F: ACCCTGGGTGCCATCTTGAG R: TCTTGTGGAAACGCCGAGAATA	146
*PGL*	NM_174179.2	F: GGCTGAGGACTACGCCAAGAA R: CACCCAGAATCAGCAGGTCAA	157
*CPT1α*	FJ415874.1	F: TCGCGATGGACTTGCTGTATA R: CGGTCCAGTTTGCGTCTGTA	100
*PPARα*	FJ415874.1	F: CATAACGCGATTCGTTTTGGA R: CGCGGTTTCGGAATCTTCT	102
*THRA*	NM_001046329.1	F:GTCAACCACCGCAAACAC R:ACAACATGCACTCCGAGAA	134
*THRB*	XM_024986440.1	F:GGAAGCAGAAGCGGAAGT R:CACATGGCAGCTCACAAA	112
*DIO1*	NM_001122593.2	F: TGGGGTAGACACAATGACGAA R: GGCCAGATTTACCCTTGTAGGA	106
*DIO2*	NM_001010992.7	F: CCACCTTCTGGACTTTGCCA R: GGAAGTCAGCCACGGATGAG	134
*DIO3*	NM_001010993.3	F: TCACTCCCTGAGGCTCTG R: CCCAGTAAATGCTTACGGATG	120
*GAPDH*	NM_001034034.2	F: GGGTCATCATCTCTGCACCT R: GGTCATAAGTCCCTCCACGA	176

*SREBP1*, sterol regulatory element-protein 1; *G6PC*, Glucose-6-phosphatase; PCK1, phosphoenolpyruvate carboxykinase 1; *ACC*, acetyl-CoA carboxylase alpha; *ACSL1*, acyl CoA synthetase 1; *ATGL*, adipose triglyceride lipase; *HSL*, hormone-sensitive lipase; *GLUT1*, solute carrier family 2 member 1; *HK1*, hexokinase 1; *PGL*, succinate dehydrogenase complex subunit D; *CPT1α*, carnitine palmitoyltransferase 1A; *PPARα*, peroxisome proliferator activated receptor alpha; *THRA*, thyroid hormone receptor alpha; *DIO1*, iodothyronine deiodinase; *GAPDH*, glyceraldehyde phosphate dehydrogenase.

## References

[1] Keunen JE, Plaizier JC, Kyriazakis L (2002). Effects of a subacute ruminal acidosis model on the diet selection of dairy cows. J Dairy Sci.

[2] Metre D, Tyler JW, Stehman SM (2000). Diagnosis of enteric disease in small ruminants. Vet Clin North Am Food Anim Pract.

[3] Enemark J, Jrgensen RJ, Kristensen NB (2004). An evaluation of parameters for the detection of subclinical rumen acidosis in dairy herds. Vet Res Commun.

[4] Plaizier JC, Krause DO, Gozho GN, Mcbride BW (2008). Subacute ruminal acidosis in dairy cows: the physiological causes, incidence and consequences. Vet J.

[5] Zhao FQ, Keating AF (2007). Expression and regulation of glucose transporters in the bovine mammary gland. J Dairy Sci.

[6] Al-Trad B, Wittek T, Penner GB (2010). Expression and activity of key hepatic gluconeogenesis enzymes in response to increasing intravenous infusions of glucose in dairy cows. J Anim Sci.

[7] Dong H, Wang S, Jia Y (2013). Long-term effects of subacute ruminal acidosis (SARA) on milk quality and hepatic gene expression in lactating goats fed a high-concentrate diet. PLoS One.

[8] Phillips CM, Goumidi L, Bertrais S (2010). Gene-nutrient interactions with dietary fat modulate the association between genetic variation of the ACSL1 gene and metabolic syndrome. J Lipid Res.

[9] Dong HB, Sun LL, Cong RH (2017). Changes in milk performance and hepatic metabolism in mid-lactating dairy goats after being fed a high concentrate diet for 10 weeks. Animal.

[10] Dann HM, Drackley JK (2005). Carnitine palmitoyltransferase I in liver of periparturient dairy cows: effects of prepartum intake, postpartum induction of ketosis, and periparturient disorders. J Dairy Sci.

[11] Xu T, Tao H, Chang G, Zhang K, Xu L, Shen X (2015). Lipopolysaccharide derived from the rumen down-regulates stearoyl-CoA desaturase 1 expression and alters fatty acid composition in the liver of dairy cows fed a high-concentrate diet. BMC Vet Res.

[12] Cronjé PB (2000). Ruminant physiology: digestion, metabolism, growth, and reproduction.

[13] Jia YY, Wang SQ, Ni YD, Zhang YS, Zhuang S, Shen XZ (2014). High concentrate-induced subacute ruminal acidosis (SARA) increases plasma acute phase proteins (APPs) and cortisol in goats. Animal.

[14] Hultquist KM, Clapper JA, Casper DP (2019). Short communication: Feeding a rumen-degradable amino acid affects plasma thyroxine and triiodothyronine concentrations. J Dairy Sci.

[15] Mullur R, Liu YY, Brent GA (2014). Thyroid hormone regulation of metabolism. Physiol Rev.

[16] Arrojo EDR, Fonseca TL, Werneck-de-Castro JP, Bianco AC (2013). Role of the type 2 iodothyronine deiodinase (D2) in the control of thyroid hormone signaling. Biochim Biophys Acta Gen Subj.

[17] Lakshmanan M, Goncalves E, Pontecorvi A, Robbins J (1992). Differential effect of a new thyromimetic on triiodothyronine transport into myoblasts and hepatoma and neuroblastoma cells. Biochim Biophys Acta Mol Cell Res.

[18] Flamant F, Gauthier K (2013). Thyroid hormone receptors: the challenge of elucidating isotype-specific functions and cell-specific response. Biochim Biophys Acta Gen Subj.

[19] Beckett GJ, Russell A, Nicol F, Sahu P, Wolf CR, Arthur JR (1992). Effect of selenium deficiency on hepatic type I 5-iodothyronine deiodinase activity and hepatic thyroid hormone levels in the rat. Biochem J.

[20] Knegsel ATMv, Brand HVD, Dijkstra J, Tamminga S, Kemp B (2005). Effect of dietary energy source on energy balance, production, metabolic disorders and reproduction in lactating dairy cattle. Reprod Nutr Dev.

[21] Guo J, Chang G, Zhang K (2017). Rumen-derived lipopolysaccharide provoked inflammatory injury in the liver of dairy cows fed a high-concentrate diet. Oncotarget.

[22] Overton TR, Drackley JK, Ottemann-Abbamonte CJ, Beaulieu AD, Emmert LS, Clark JH (1999). Substrate utilization for hepatic gluconeogenesis is altered by increased glucose demand in ruminants. J Anim Sci.

[23] Aschenbach JR, Kristensen NB, Donkin SS, Hammon HM, Penner GB (2010). Gluconeogenesis in dairy cows: the secret of making sweet milk from sour dough. IUBMB Life.

[24] Xu T, Tao H, Chang G, Zhang K, Xu L, Shen X (2015). Lipopolysaccharide derived from the rumen down-regulates stearoyl-CoA desaturase 1 expression and alters fatty acid composition in the liver of dairy cows fed a high-concentrate diet. BMC Vet Res.

[25] Graugnard DE, Berger LL, Faulkner DB, Loor JJ (2010). High-starch diets induce precocious adipogenic gene network up-regulation in longissimus lumborum of early-weaned Angus cattle. Br J Nutr.

[26] Suh JH, Sieglaff DH, Zhang A (2013). SIRT1 is a direct coactivator of thyroid hormone receptor beta1 with gene-specific actions. PLoS One.

[27] Park EA, Song S, Vinson C, Roesler WJ (1999). Role of CCAAT enhancer-binding protein beta in the thyroid hormone and cAMP induction of phosphoenolpyruvate carboxykinase gene transcription. J Biol Chem.

[28] Sinha RA, Singh BK, Yen PM (2014). Thyroid hormone regulation of hepatic lipid and carbohydrate metabolism. Trends Endocrinol Metab.

[29] Heitzman RJ, Hibbitt KG, Mather I (1971). The effects of thyroxine on hepatic gluconeogenesis and ketogenesis in dairy cows. Eur J Biochem.

[30] Tian P, Luo Y, Li X (2017). Negative effects of long-term feeding of high-grain diets to lactating goats on milk fat production and composition by regulating gene expression and DNA methylation in the mammary gland. J Anim Sci Biotechnol.

[31] Li L, Cao Y, Xie Z, Zhang Y (2017). A high-concentrate diet induced milk fat decline via glucagon-mediated activation of AMP-activated protein kinase in dairy cows. Sci Rep.

[32] Lévesque J, Dion S, Brassard M, Rico D, Gervais R, Chouinard Y (2018). PSXV-24 Dietary strategies to reduce the impact of high-concentrate diet on performance, ruminal fermentation and milk composition of dairy goats. J Anim Sci.

[33] Zebeli Q, Dunn SM, Ametaj BN (2011). Perturbations of plasma metabolites correlated with the rise of rumen endotoxin in dairy cows fed diets rich in easily degradable carbohydrates. J Dairy Sci.

[34] Jiang X, Zeng T, Zhang S, Zhang Y (2013). Comparative proteomic and bioinformatic analysis of the effects of a high-grain diet on the hepatic metabolism in lactating dairy goats. Plos One.

[35] Capuco AV, Connor EE, Wood DL (2008). Regulation of mammary gland sensitivity to thyroid hormones during the transition from pregnancy to lactation. Exp Biol Med (Maywood).

[36] Oppenheimer JH (1985). Thyroid hormone action at the nuclear level. Ann Intern Med.

